# Nanosecond Transient IR Spectroscopy of Halorhodopsin in Living Cells

**DOI:** 10.1021/jacs.4c03891

**Published:** 2024-07-01

**Authors:** Sabine Oldemeyer, Mariafrancesca La Greca, Pit Langner, Karoline-Luisa Lê Công, Ramona Schlesinger, Joachim Heberle

**Affiliations:** † Experimental Molecular Biophysics, Department of Physics, Freie Universität Berlin, Arnimallee 14, 14195 Berlin, Germany; ‡ Genetic Biophysics, Department of Physics, Freie Universität Berlin, Arnimallee 14, 14195 Berlin, Germany

## Abstract

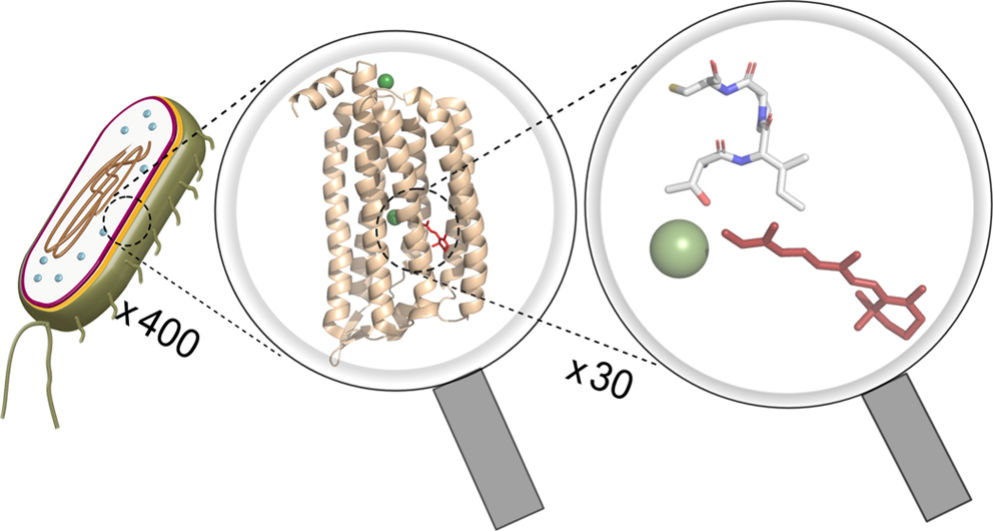

The ability to track minute changes of a single amino acid residue in a cellular environment is causing a paradigm shift in the attempt to fully understand the responses of biomolecules that are highly sensitive to their environment. Detecting early protein dynamics in living cells is crucial to understanding their mechanisms, such as those of photosynthetic proteins. Here, we elucidate the light response of the microbial chloride pump *Nm*HR from the marine bacterium *Nonlabens marinus*, located in the membrane of living *Escherichia coli* cells, using nanosecond time-resolved UV/vis and IR absorption spectroscopy over the time range from nanoseconds to seconds. Transient structural changes of the retinal cofactor and the surrounding apoprotein are recorded using light-induced time-resolved UV/vis and IR difference spectroscopy. Of particular note, we have resolved the kinetics of the transient deprotonation of a single cysteine residue during the photocycle of *Nm*HR out of the manifold of molecular vibrations of the cells. These findings are of high general relevance, given the successful development of optogenetic tools from photoreceptors to interfere with enzymatic and neuronal pathways in living organisms using light pulses as a noninvasive trigger.

## Introduction

A potent tool to investigate structure–function relationships in biological samples even at the level of individual amino acids is presented by light-induced difference Fourier transform infrared (FTIR) spectroscopy.^1,2^ Due to the strong background absorption, proteins are usually studied by IR difference spectroscopy isolated from their native environment, the living cell, but exposed to an artificial aqueous environment. Thus, the interpretation of spectroscopic results recorded on isolated proteins may be compromised in the absence of the biological context. Working with purified membrane proteins presents the challenge of recreating a stabilizing environment using detergents, lipids, or nanodiscs, which can differ substantially from in vivo conditions, potentially affecting protein mechanisms.

To overcome the drawbacks of this approach, various in-cell spectroscopic techniques, such as UV/vis, fluorescence, NMR, and electron paramagnetic resonance (EPR) spectroscopy, have been developed in the past.^3−6^ In-cell infrared difference spectroscopy, for example, which overcomes the limitation of probing mainly the changes in the chromophore but rather the whole protein including amino acids and secondary structural elements, has recently been successfully established.^7^ However, this technique has only been applied to soluble proteins and has been limited to experiments in the steady-state transmission or attenuated total reflection (ATR) configuration. In another study, intact rod cells were used to study the light response of the photoreceptor rhodopsin using time-resolved IR difference spectromicroscopy.^8^ The low temporal resolution provided only limited insight into the dynamic processes and the details of the photocycle. Similarly, studies on living cells of the unicellular algae *Chlamydomonas reinhardtii* via infrared spectromicroscopy lacked sufficient time resolution for a more detailed analysis.^9^ Notably, picosecond time-resolved studies in HeLa tumor cells were performed recently, delivering information on their potential use as photodynamic therapeutic agents.^10^

**Figure 1:**
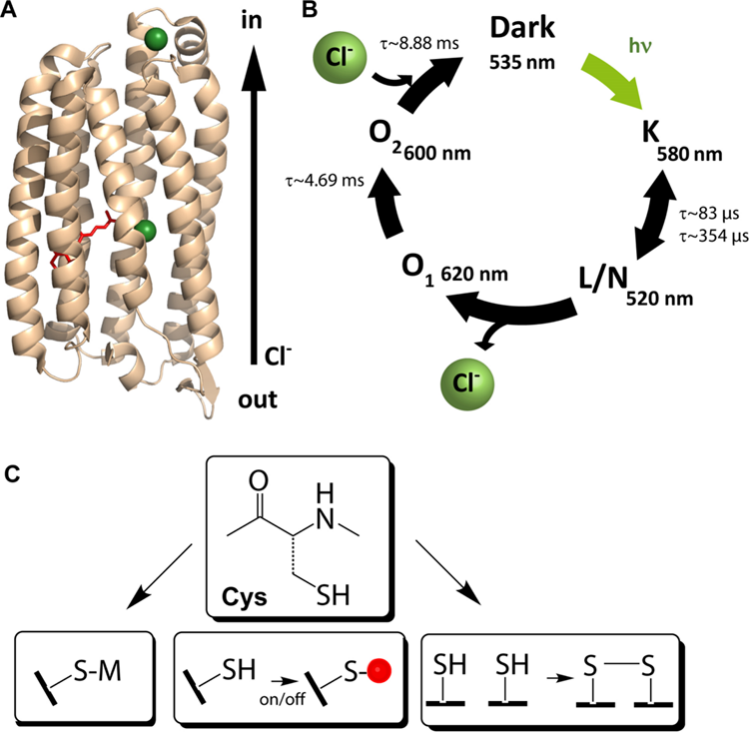
(A) Dark-state structure of *Nm*HR with the retinal chromophore (red) and bound chloride ions (green) (PDB: 5G28(16)). The pumping direction is indicated with an arrow. (B) Model of the *Nm*HR photocycle as derived from time-resolved UV/vis experiments.^14^ (C) Sketch of different functional roles of cysteine residues such as metal binding, regulatory purposes, and disulfide bridge formation.

Given that transmembrane retinal proteins are at the forefront of optogenetic tools, advances in this field are of major importance for medical applications.^11^ Some members of the retinal family are inward-directed halide-pumping rhodopsins, such as the halorhodopsins found in halophilic archaea.^12,13^ A lineage distinct from the archaeal light-driven Cl^–^-pumping rhodopsins is represented by the halorhodopsin *Nm*HR from the Gram-negative marine bacterium *Nonlabens marinus* (Figure 1A). *Nm*HR binds a retinal chromophore in the all-*trans* configuration, which initiates the transport cycle upon photoisomerization. The dynamics of the light response of the chloride pump have been studied spectroscopically in the visible and infrared range on purified samples,^14,15^ and a photocycle has been established (Figure 1B).

Within the group of the 20 canonical amino acids, cysteine (Cys) residues are among the rarest but most highly conserved ones.^17^ They are predominantly located at essential functional sites of proteins and play a vital role in various processes, including redox chemistry, redox sensing, metal binding, and catalysis regulation^18−20^ (Figure 1C). In Alzheimer’s pathogenesis, disulfide bridge formation is essential for peptide oligomerization and, therefore, ultimately responsible for the formation of amyloid-β peptides.^21^

Cysteine residues show an extreme pattern of conservation, suggesting a strong selective pressure to retain the residues at functionally important sites.^22^ In humans, mutations of Cys are the cause of genetic diseases more often than predicted on the basis of their abundance, emphasizing the functional importance of this amino acid.^23^ The prominent role of Cys is rationalized by the fact that the residue represents not only one of the two sulfur-containing amino acids but also harbors the sulfur in a reactive thiol or “sulfhydryl” group, which can act as a nucleophile in enzymatic catalysis and is redox active. The p*K*_a_ of the terminal thiol in surface-exposed cysteines was determined to be around 8.0^24^ and is, therefore, close to neutral, rendering cysteines suitable donors and acceptors in proton transfer reactions.

Due to the versatile involvement of cysteine residues in crucial biological processes,^25^ it is essential to monitor their reaction to gain a complete understanding of the mechanism under study. Particularly for light-sensing proteins, IR spectroscopy provides excellent spectral and temporal resolution with the benefit of minimal interference with the system under investigation due to the low energy of the probe light.^26,27^

A major challenge for the application to biological samples is the strong absorption of water in the IR region. Here, the S–H stretching vibrations of cysteine residues are unique as they absorb in the spectral window between 2600 and 2500 cm^–1^ where hardly any other biologically relevant vibrational bands appear.^28−30^ The terminal S–H group of cysteines is very sensitive to changes in H-bonding within proteins, making it possible to track minute changes in the immediate environment of the amino acid side chain.^27^ C105 of *NmH*R is located close to the functionally relevant NTQ motif (N98, T102, and Q109).^31^ Even though the involvement of cysteine residues in the photocycle of several other retinal proteins has been reported,^29,32−34^ a possible role of C105 in the photocycle of *Nm*HR has not been uncovered yet.

The strong IR emission of the quantum cascade laser (QCL) allows the recording of ns time-resolved infrared data of *Nm*HR residing in living *Escherichia coli* cells. By tracking the early changes in the light response of *Nm*HR, we discovered the deprotonation of a single cysteine residue within 75 ns after the light activation in the environment of living cells. These results underline the high potential of the method for monitoring the reaction dynamics of cysteine residues in a cellular environment.

## Materials and Methods

### Protein Expression and Purification

The gene of *Nm*HR (kindly provided by Dr. Przemyslaw Nogly, ETH Zurich), with an additional 10xHis-tag at the C-terminus, was cloned into pET27b. The plasmid was transformed into *E. coli* strain BL21-CodonPlus (DE3)-RP (Stratagene), and the cells were grown at 37 °C in brain heart infusion (BHI) medium with 50 μg/mL kanamycin. Further steps of the protein expression and purification were carried out similarly to previous protocols.^16^

The expression protocol was modified for the whole-cell samples to reduce the expression level. Cells were grown to an optical density of 4.0 before the induction with 0.5 mM isopropyl β-d-1-thiogalactopyranoside (IPTG) and the addition of 50 μM retinal. The temperature was reduced to 30 °C, and the cells were harvested after 2 h.

C55S and C105S single-point mutations were introduced via Quick-change mutagenesis polymerase chain reaction (PCR). The designed primers were: 5′-GCA ACT GCG CTT TCT TCT ATT GTT ATG GTA TCT GC-3′ and 5′-GCA GAT ACC ATA ACA ATA GAA GAA AGC GCA GTT GC-3′ for *Nm*HR C55S and 5′-GAT GGC GAC GAT TCC TTC TTT ATT GTT ACA ATT ATT GAT TG-3′and 5′-CAA TCA ATA ATT GTA ACA ATA AAG AAG GAA TCG TCG CCA TC-3′ for *Nm*HR C105S. The resulting plasmids were verified by sequencing, and the subsequent expression and purification of the mutated proteins were done by the same protocol as before for the wild-type (WT) protein. To obtain the whole-cell samples, the protein was not extracted from the cells after expression. Instead, 45 mL of the cell suspension were centrifuged (10 min, 4000*g*) and washed three times with 150 mM NaCl and 5 mM KCl according to an established protocol.^7^

### Time-Resolved UV/Vis Spectroscopy

Time-resolved UV/vis spectroscopy on *Nm*HR solubilized in detergent was conducted on protein in 0.03% *n*-dodecyl-β-d-maltoside (DDM) in 20 mM *N*-(2-hydroxyethyl)piperazine-*N*′-ethanesulfonic acid (HEPES) and 0.1, 0.4, 1, and 4 M NaCl at pH 7.5. A solution of OD_535_ = 0.6 was prepared in a quartz cuvette with an optical path length of 1 cm. The whole-cell sample was suspended in the 150 NaCl mM and 5 mM KCl washing solution and spread out on a BaF_2_ window. For the fully hydrated samples, the cell suspension was immediately sealed with a second window. To generate a sample with reduced water content, the cells were gently dried under atmospheric pressure for 30 min before sealing them with a second BaF_2_ window. A commercially available setup (LKS80, Applied Photophysics) previously described^35^ was used for the UV/vis flash photolysis experiments. Pulsed excitation of the samples at 532 nm was accomplished by a Nd:YAG laser (Quanta-Ray, Spectra-Physics) in combination with an optical parametric oscillator. The excitation energy density was set to 3 or 5 mJ/cm^2^ for detergent-solubilized proteins and whole-cell samples, respectively. For the purified protein, 10 kinetic traces were measured for each wavelength and subsequently averaged. For the whole-cell suspension, 50 averages per wavelength were recorded. The time between each excitation was set to 1 s to ensure the recovery of the initial ground state.

### FTIR Spectroscopy

For the preparation of detergent-solubilized samples for IR experiments, 4 μL of a highly concentrated (∼70 mg/mL) solution of *Nm*HR in 150 mM NaCl and 20 mM HEPES at pH 7.5 with 0.03% DDM was placed on a BaF_2_ window. The water content of the sample was gently reduced by letting the sample open for 5 min before sealing it with a second BaF_2_ window. A ratio of the amide I to amide II band of 2.3–2.5 confirmed the sufficient hydration of the samples.^36^ For the whole-cell samples, 8 μL of the cell suspension was placed on a BaF_2_ window and left open for up to 30 min to reduce the extracellular water content.

Millisecond time-resolved spectra were recorded using a Bruker 80v FTIR spectrometer in the rapid scan mode running at a spectral resolution of 4 cm^–1^. For spectra covering the amide and retinal bands, the spectral range was scanned from 1975–0 cm^–1^ single-sided, with a scanner velocity of 280 kHz, using a low pass optical filter with a cutoff of ∼1900 cm^–1^ to avoid aliasing. After pulsed excitation (1 Hz) of the samples under conditions comparable to the time-resolved UV/vis experiments (vide supra), spectra were recorded with a time resolution of ∼11 ms. This process was repeated 1000 times for the whole-cell sample to achieve a sufficient signal-to-noise ratio (SNR). About 6000 scans were averaged for *Nm*HR solubilized in detergent. To obtain rapid scan spectra covering the cysteine range around 2600–2500 cm^–1^, the spectral range was restricted from 3950 to 0 cm^–1^ using a low pass optical filter with a cutoff of ∼3990 cm^–1^. The scanner velocity was set to 240 kHz, and 7 × 1000 and 3 × 1000 scans were averaged for detergent and whole-cell samples, respectively.

### Time-Resolved IR Spectroscopy Using Tunable QCLs

Time-resolved experiments in the nanosecond to millisecond time regime were conducted using a home-built quantum cascade laser (QCL) setup as previously described in ref (37). The setup allows a time resolution of up to 15 ns. Briefly, the sample was excited by a pulsed visible laser at 532 nm, and IR transients are recorded at a specific frequency for a given number of repetitions. To detect spectral information between 1560–1516 and 2600–2200 cm^–1^, two different QCLs were employed and the frequency range was sampled in steps of 2 cm^–1^. To avoid the excitation of photocycle intermediates, *Nm*HR was excited with a repetition rate of 1 Hz. Pulsed laser excitation and preparation of the sample was similar to the samples used for rapid scan, but a higher volume of 6 μL for the purified *Nm*HR sample and 20 μL of the whole-cell sample was used to improve SNR. An even higher amount of sample (8 and 35 μL for purified and whole cells, respectively) was used in the spectral range between 2600 and 2200 cm^–1^, given the fact that the sample is almost transparent in this frequency region. Experiments on whole-cell samples in the region of 1560–1520 cm^–1^ were repeated 1300 times in total, with the first measurement of 650 scans per wavenumber running from lower to upper wavenumbers and the second run in reverse. For the detergent-solubilized sample, 250 scans were averaged. The whole-cell sample was averaged 720 times in the spectral region of 2578–2526 cm^–1^. For the experiment on *Nm*HR solubilized in detergent, 100 scans were averaged.

### Data Analysis

The time-resolved data sets acquired from the QCL experiments in the spectral range from 2600 to 2500 cm^–1^ were subjected to singular value decomposition (SVD) analysis, baseline correction, and smoothing using MatLab-based scripts.^38^ Three and five SVD components were selected for the whole-cell samples and detergent-solubilized proteins, respectively. SVD analysis was performed to reduce noise levels.

## Results

*Nm*HR was overexpressed in *E. coli*, and cells were harvested. The cell pellet was gently washed, and the purple color of the pellet indicated successful expression (Figure S1). The viability of the cells before and after the experiments was confirmed by a viability assay (Figure S2).

To test whether *Nm*HR exhibits a photocycle in the environment of whole cells, transient UV/vis flash photolysis data were recorded at 650 nm, a wavelength indicative of the rise of the O intermediate.^14^ Due to the very similar spectral and kinetic characteristics of the O_1_ and O_2_ states, we will not distinguish between the two O states in the following text. A comparison of cells with and without induced expression revealed that only the cells expressing *Nm*HR showed the rise and decay of the absorbance at 650 nm after light excitation in UV/vis flash photolysis experiments (Figure S3). Thus, the detected signal arises from photoactivated *Nm*HR and not from other cellular components.

**Figure 2:**
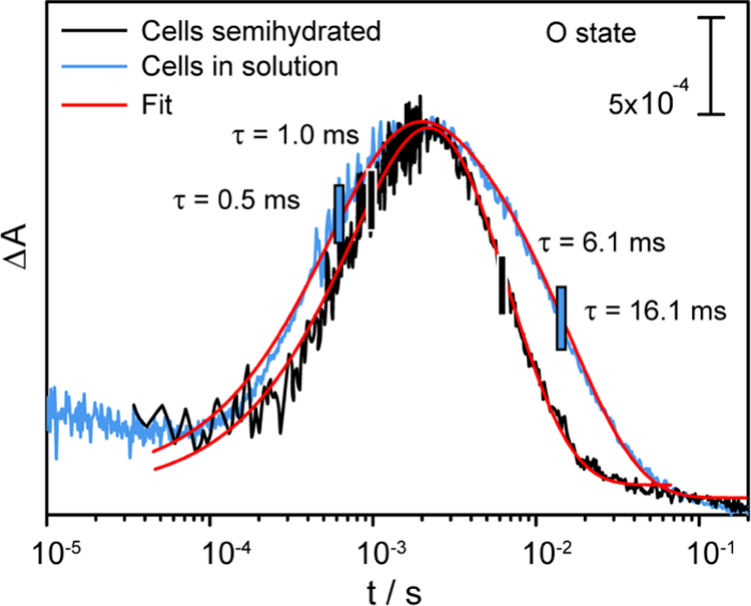
Time-resolved UV/vis flash photolysis data of *Nm*HR WT in cells detected at 600 nm. Cells overexpressing *Nm*HR either in solution (black) or in semihydrated after drying for 30 min (blue) are compared. The trace of the sample with reduced water content was scaled by a factor of 0.55. Kinetic traces show the characteristic rise and decay of the O intermediate with indicated time constants.

The kinetics of the whole cells overexpressing *Nm*HR (*Nm*HR-cells) in two different sample preparations were compared to detergent-solubilized *Nm*HR (in 4 M, 1 M, 400 mM, and 150 mM NaCl, pH 7.5) to test if the reduction of the water content hampers the photocycle (for details, see Materials and Methods). In one sample, the extracellular water content was gently reduced by drying under atmospheric pressure for 30 min (semihydrated *Nm*HR-cells), while the other sample was fully hydrated in a 150 mM NaCl/5 mM KCl solution. For both cell samples, kinetic traces were recorded at 500, 580, 600, and 650 nm (Figure S4, Table S1). The data at 600 nm, indicative of the O state, during which a new chloride ion is taken up by the protein, was compared to the time constants of the detergent-solubilized sample (Figure 2, Table 1, Figure S5). The kinetic analysis of the transients revealed an acceleration of the decay from 16.1 to 6.1 ms in samples with reduced extracellular water content (Table 1). The acceleration can be rationalized with the higher salt concentration in the dried sample as chloride uptake is facilitated at increased NaCl concentration as reported previously^14^ and confirmed in purified proteins (Table 1, Figure S5). The semihydrated cells show dynamics similar to those observed in the detergent-solubilized sample in 4 M NaCl, which exhibits a decay constant of 8.3 ms of the O state. Due to other factors influencing the decay constant, such as the hydration level and lipid environment, it is not possible to draw direct conclusions about the intracellular NaCl concentration. Still, our results confirm the functionality of the protein in both cell preparations.

**Table 1:** Time Constants of the Rise and Decay of the O State of *Nm*HR in Fully Hydrated Cells, Semihydrated Cells, and Detergent-Solubilized *Nm*HR Monitored at 600 nm (For Full Kinetic Traces of Detergent-Solubilized *Nm*HR, See Figure S5)

600 nm	*Nm*HR-cells, in solution, 150 mM NaCl (ms)	*Nm*HR-cells, semihydrated (ms)	detergent-solubilized 150 mM NaCl (ms)	detergent-solubilized 4 M NaCl (ms)
τ_rise_	0.5	1.0		0.4
τ_decay_	16.1	6.1	18.3	8.3

**Figure 3:**
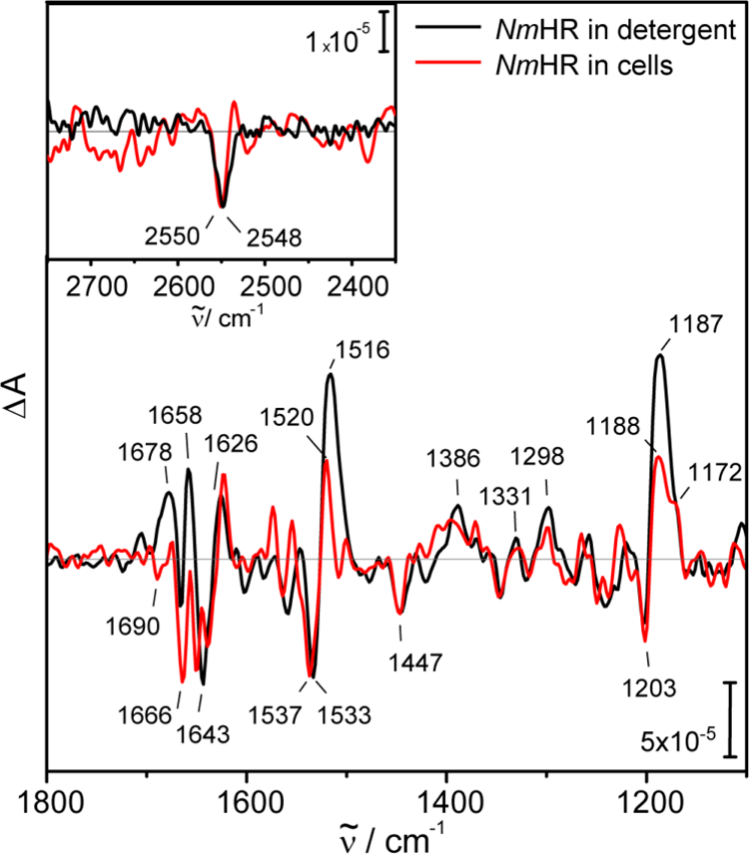
Rapid scan FTIR spectra of *Nm*HR wild-type overexpressed in whole cells, under semidried conditions (red) and solubilized in detergent at pH 7.5, 150 mM NaCl (black). The samples were excited with a pulsed laser at 532 nm for 10 ns every second. Spectra at ∼11 ms show a predominant contribution of the O state. Inset: Spectra of the cysteine region at 11 ms. The spectrum of the detergent-solubilized sample is scaled by a factor of 0.8.

As visible spectroscopy probes electronic changes in the retinal chromophore exclusively, we performed time-resolved FTIR spectroscopy to record vibrational changes indicative of structural changes in the surrounding protein. The spectral range between 1800 and 1100 cm^–1^ was monitored, covering relevant vibrational bands such as the C = C stretching modes of the retinal chromophore, the amide I and II bands of the peptide backbone, and the C=N vibrations of the retinal Schiff base (RSB) (Figure 3). Light-induced difference spectra of detergent-solubilized *Nm*HR and intact *E. coli* cells overexpressing *Nm*HR were recorded in transmission mode with a time resolution of ∼11 ms (Figure 3). The light-induced difference spectrum of *Nm*HR in detergent (black) exhibits negative marker bands at 1666, 1643, 1533, 1447, and 1203 cm^–1^. These vibrational bands agree well with the corresponding bands found in the light-induced spectra of related microbial retinal proteins.^39,40^ Characteristic positive bands are detected at 1658, 1626, 1516, 1331, 1298, and 1187 cm^–1^ with the complete band assignment provided in Table S2. The light-induced difference spectrum of *Nm*HR in the whole-cell sample shows very similar vibrational signatures as compared to the sample in detergent. Differences can be observed at the amide I region, possibly due to the higher rigidity of the cell wall as compared to detergent micelles, resulting in increased lateral pressure. At the frequency of the C=C stretching vibration of the retinal, the band pattern is slightly upshifted by 4 cm^–1^ to 1537 (−) and 1520 (+) cm^–1^. As previously reported, this shift can be related to the different salt concentrations of the samples, which cause a shift in the electronic spectrum (Supporting Information SI Figure 6).^14,41^ The vibrational modes representing the retinal C–C stretching are found at 1203 (−) cm^–1^, 1188 (+) cm^–1^, and a shoulder at 1172 (+) cm^–1^. The appearance of the shoulder at 1172 cm^–1^ in the cell samples arises from a slightly different mixture of intermediates formed at this time point with a higher contribution of the O state as compared to the sample in detergent.^40^ Overall, the vibrational bands described are in good agreement with bands previously assigned to the O state, confirming the successful detection of ms-time-resolved light-induced IR spectra of *Nm*HR in the living cells.

### Cysteine Vibrations

Previous studies on retinal proteins have reported the involvement of cysteine residues in the photoreaction.^29,32−34,42^ To investigate the possible involvement of a cysteine in the light response of *Nm*HR, the characteristic region for S–H stretching vibrations from 2600 to 2500 cm^–1 ^^43^ was probed. In the detergent-solubilized protein, a prominent negative band was detected at 2548 cm^–1^, indicating the deprotonation of a cysteine (Figure 3 inset). In the whole-cell sample, the same band was found, slightly shifted to 2550 cm^–1^, showing that this cysteine is also deprotonated as part of the light response of *Nm*HR in the environment of a living cell.

To learn more about the earlier states and the dynamics of the photocycle, spectra with microsecond time resolution were acquired. Due to the high intensity of the QCL emission, this technique also enables the use of thicker samples, making it ideal for studying whole cells. Kinetic traces of the detergent-solubilized and the whole-cell samples were recorded after pulsed laser excitation at 532 nm in the region of 1516–1560 cm^–1^, covering the C=C stretching modes of the retinal chromophore.

**Figure 4:**
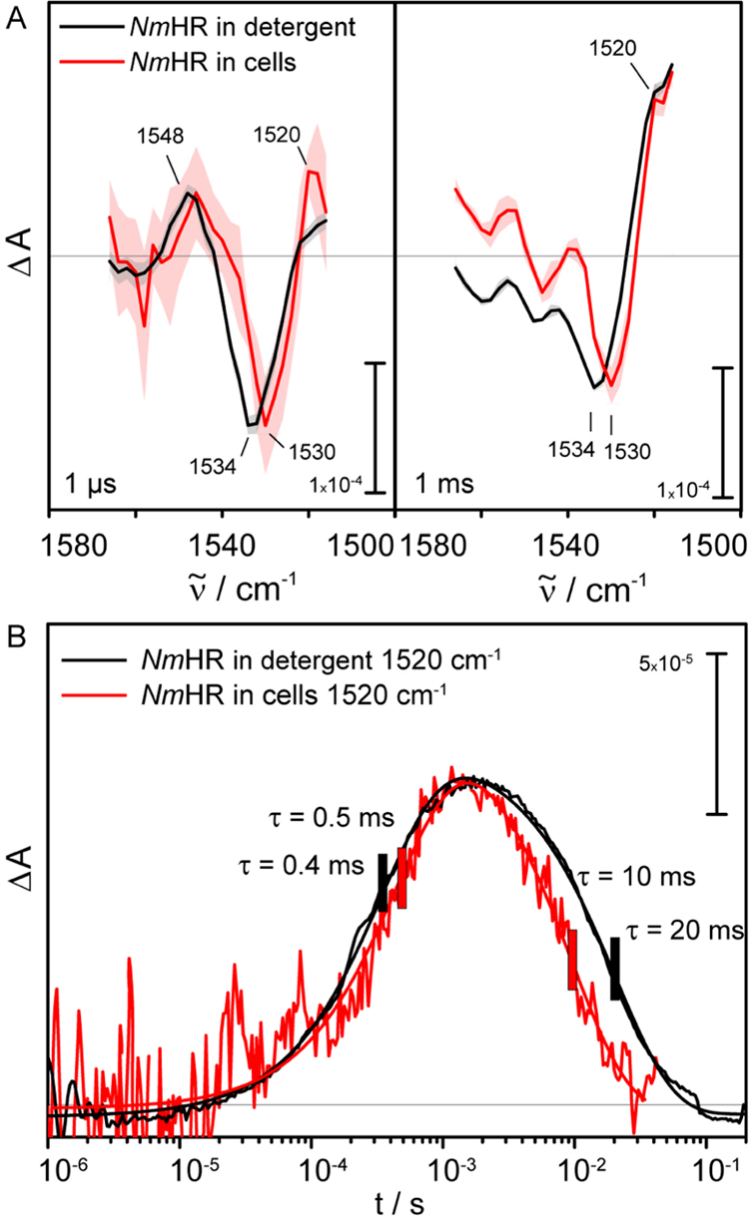
Time-resolved IR spectroscopy on *Nm*HR wild-type overexpressed in whole cells, under semidried conditions (red) and solubilized in detergent at pH 7.5, 150 mM NaCl, (black). (A) Spectra extracted at 1 μs and 1 ms, before and upon maximum accumulation of the O state, respectively. The shaded area indicates the standard deviation times two. Spectra from detergent samples are multiplied by a factor of 0.0742. (B) Kinetic traces extracted at 1520 cm^–1^. The kinetic trace of the detergent-solubilized sample is multiplied by a factor of 0.055.

The spectrum at 1 μs (Figure 4A) shows the negative band mainly caused by the bleaching of the ethylenic mode of the dark state, at 1534 and 1530 cm^–1^ for the detergent-solubilized *Nm*HR and the whole-cell sample, respectively. Positive bands at 1548 and 1551 cm^–1^ can be assigned to the L(N) state, which was found to have slightly blue-shifted absorbance in the visible.^14^ Interestingly, the bands at 1534 and 1530 cm^–1^ in detergent and whole cells, respectively, are much less intense and much broader at 1 ms compared to the spectrum obtained at 1 μs. This indicates considerable spectral overlap between the bands of the dark state and the O intermediate present at 1 ms. As the O state rises at 1520 cm^–1^, its shoulder overlaps and partially cancels out the retinal bleach band, thereby reducing its intensity. As for the broadening of the ethylenic mode of the dark state, it is most likely due to the decay of the L(N) intermediate,^39^ which gave rise to the band at 1548 cm^–1^. The kinetic trace at 1520 cm^–1^ allows us to follow the dynamics of the O state (Figure 4B) and shows an almost simultaneous rise of the intermediate in both samples with time constants of 0.4 and 0.5 ms, respectively,^15^ which are in very good agreement with UV/vis data.^14^ The decay constants of 10 and 20 ms, respectively, are consistent with the decay of the O state.

Given that the rapid scan spectra at 11 ms showed a negative band in the spectral region known for S–H vibrations (Figure 3, inset), the residue responsible for the signal was identified via mutational studies. *Nm*HR contains only two cysteines, C55 and C105. The C105S and C55S variants were expressed, purified, and analyzed via light-induced FTIR difference spectroscopy (Figures S7 and S8). Both variants showed a photocycle, but the negative band was retained only in the spectra of the C55S mutant (Figure S7). Thus, C105, close to the threonine 102 of the NTQ motive, is the reactive cysteine. The C105S variant was, therefore, included in the analysis as a negative control.

**Figure 5:**
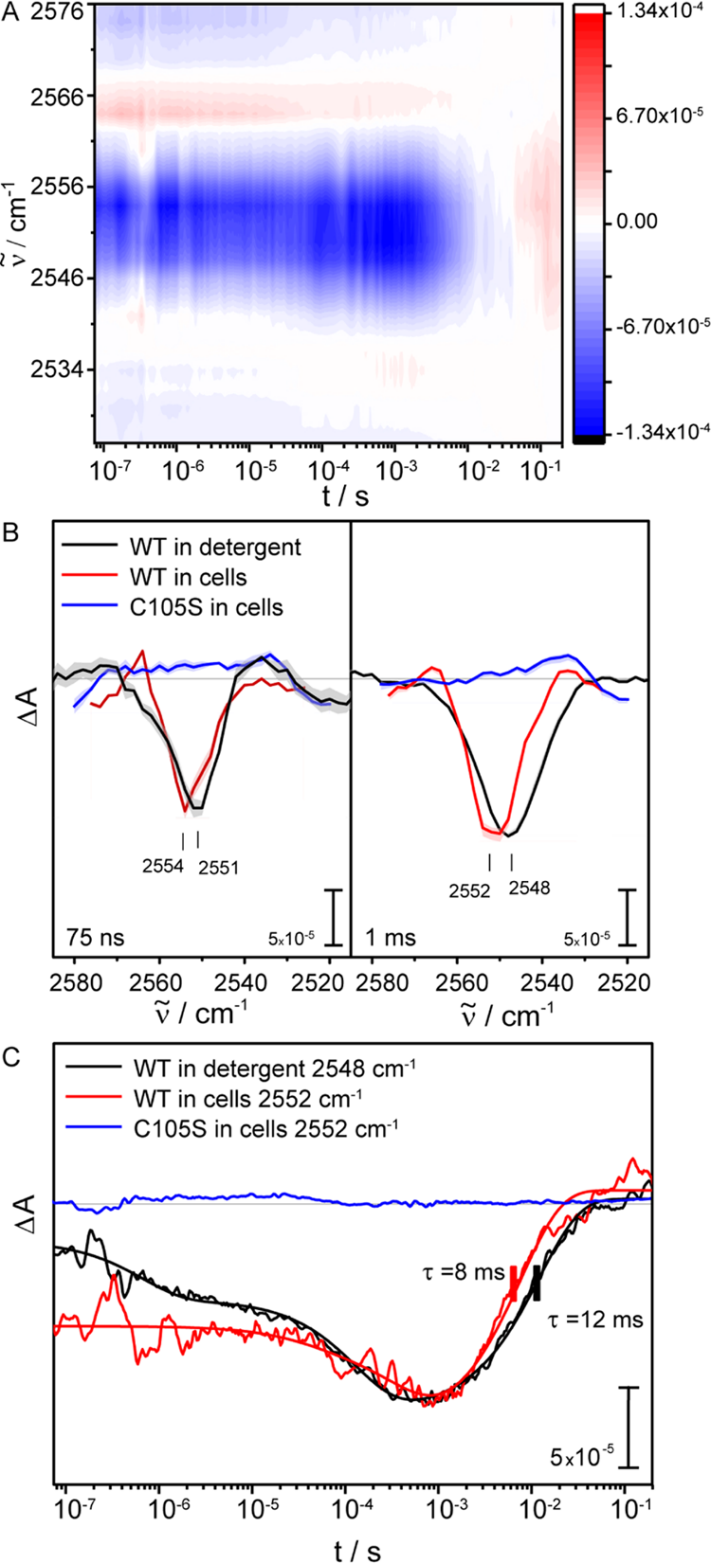
Time-resolved IR spectroscopy on *Nm*HR WT overexpressed in whole cells, under semidried conditions (red), solubilized in detergent at pH 7.5, 150 mM NaCl (black), and the *Nm*HR C105S mutant in cells (blue). (A) Heat map of *Nm*HR overexpressed in cells. (B) Spectra of *Nm*HR WT in cells, detergent, and the C105S mutant in cells extracted at 75 ns and 1 ms, before and after the maximum accumulation of the O state, respectively. Spectra of detergent samples are multiplied by a factor of 0.65 and 0.34 for 75 ns and 1 ms, respectively. (C) Kinetic traces extracted at the maximum difference absorbance of the cysteine band. The kinetic trace of the detergent-solubilized sample is multiplied by a factor of 0.4.

QCL data were collected in this spectral region with nanosecond time resolution to study the dynamics of the deprotonation of the C105 (Figure 5). Cells overexpressing the C105S mutant were included as a control and the cell sample showed the light-induced bleaching of the retinal C=C stretch around 1520 cm^–1^ (Figure S8). Our home-built QCL setup was used to detect kinetics from 75 ns to 1 s in the region from 2580 to 2520 cm^–1^ (Figure 5A). For the wild-type protein in cells and solubilized in detergent, distinct negative bands at 2554 and 2551 cm^–1^, respectively, are detected at 75 ns (Figure 5B, left panel). At 1 ms, the negative band remains in both samples but shifts to lower wavenumbers at 2552 and 2548 cm^–1^ for *Nm*HR in cells and in detergent micelles, respectively, indicating a stronger hydrogen bonding of the residue (Figure 5B, right panel).^32^ The shift of the absorbance minimum at later time points indicates sample inhomogeneity, possibly due to the presence of two different cysteine rotamers with respect to the S–H torsion as previously observed in other proteins.^28,32,44,45^ The peaks at 75 ns and 1 ms of the whole-cell sample were fitted with Gaussians to verify this assumption (Figure S9). The best-fit result was obtained by using two Gaussians with maxima at 2555 and 2549 cm^–1^ for both time points, supporting the theory of two different cysteine rotamers with different deprotonation dynamics.

The kinetic traces for wild-type *Nm*HR in cells and in detergent show a negative signal at 75 ns close to the maximum time resolution of the setup. The recovery of the signal was fitted monoexponentially with 8 and 12 ms for cells and detergent, respectively (Figure 5C). The time constants are very similar to those found for the O state decay at 1520 cm^–1^ (10 and 20 ms) (Figure 4B), indicating the reprotonation of C105 just before or during ground-state recovery. The *Nm*HR C105S sample does not show the negative signal at any time as shown by the kinetic traces at 2550 cm^–1^ (blue traces in Figure 5B,C). The absence of a band in the C105S mutant in cells shows that the formation of the cysteine band in the cell sample can be traced back to overexpressed *Nm*HR in the cells. Therefore, it is not an artifact of the cells themselves in response to light activation or ion pumping.

## Discussion

We studied the light response of the microbial chloride pump *Nm*HR in the environment of living cells as compared to the purified protein using nanosecond time-resolved UV/vis and infrared spectroscopy. Employing rapid scan FTIR and a tunable quantum cascade laser, the light-induced dynamics of *Nm*HR were recorded, uncovering the deprotonation of a single cysteine residue. The deprotonation of the cysteine occurs within 75 ns, whereas the reprotonation is observed concomitantly to the recovery of the ground state with time constants of around 10 ms.

As expected for a chloride pump, the photocycle kinetics of *Nm*HR depends on the chloride concentration of the medium.^14^ In particular, the decay of the O state, which is associated with the uptake of a new chloride ion, is accelerated in detergent-solubilized samples. This phenomenon, which has been reported for detergent-solubilized *Nm*HR, appears to persist in the cellular environment, as the decay of the O state is accelerated from τ = 16.1 ms at 150 mM NaCl to τ = 6.1 ms in the semihydrated samples with a higher NaCl concentration. On the other hand, the rise of the O state was observed to show a deceleration at elevated NaCl concentrations, rationalized by a lower propensity to release the ion. The same trend was observed in the whole-cell samples at differing hydration levels which go along with altered NaCl concentrations. However, the time constant of the O state rise of τ = 0.6 ms in the cells at 150 mM NaCl is even higher than that found in a 4 M NaCl detergent sample with τ = 0.4 ms. This would indicate an intracellular chloride concentration of over 4 M in the fully hydrated cells, which seems unrealistic given that values between 90 and 210 mM NaCl have previously been reported for the intracellular NaCl concentration of *E. coli* cells.^46−48^ Only for extremely halophilic bacteria grown under extremely high salt concentrations of around 4 M, chloride concentrations of up to 4.3 M have been reported.^49^ Thus, we conclude that the delayed formation of the O state observed in the cells is most likely caused by other factors. The embedding of the protein in the bacterial cell membrane could affect the photocycle due to the induced lateral pressure, possibly impacting secondary structural changes as observed in other light-driven membrane proteins.^50,51^ Furthermore, as the cells represent closed systems, the membrane potential, possibly altered by the inward Cl^–^ pumping of *Nm*HR, might also influence the release of chloride into the cytoplasm. It is unclear whether the cell is able to regain its electrochemical potential across the membrane during the experiment when chloride is actively transported into the cell with each illumination. To define the reason for the changes in the dynamics of the photocycle intermediates of *Nm*HR in *E. coli* cells, titration experiments, in which the NaCl concentration and the hydration levels are strictly controlled, need to be performed in the future. Only if the effect of the water content and the salt concentration in the sample can be studied separately, one can distinguish the effect of those variables on the kinetics. Overall, the results of our transient spectroscopic experiments confirm the functionality of the protein in different preparations and show the same trend with changing salt concentrations. However, the interference of additional effects does not allow a quantitative analysis or estimation of intracellular chloride concentrations.

Rapid scan FTIR spectroscopy was used to show the accumulation of the O intermediate in the chloride pump *Nm*HR in detergent as well as in the environment of living *E. coli* cells at 11 ms. To our knowledge, this is the first study in which time-resolved transmission FTIR spectroscopy has been performed on a membrane protein in the environment of living cells. The spectrum shows characteristic features of the conversion of all-*trans* to 13-*cis* retinal such as negative bands at 1537 and 1203 cm^–1^ and positive features at 1187 cm^–1^, which are also found in the spectrum of the detergent-solubilized sample. Interestingly, the band patterns of the two samples are not identical but show small differences throughout the entire spectral region, indicating the presence of a slightly different mixture of intermediates accumulated at 11 ms after pulsed photoexcitation. Given the sensitivity of the protein’s photocycle to different salt concentrations, its hydration level, and its lipid environment,^14^ this finding is not surprising. In the amide I region, indicative of secondary structural changes, the signals are also present at similar frequencies in both samples. However, in *Nm*HR-cells, the intensity of the bands is lower, indicating less strong alterations in the protein structure. This could be explained by less pronounced changes in the helix C of *Nm*HR, which has been reported to be kinked in the presence of a chloride ion and to straighten as part of the light response.^15^ It can be speculated that the observed kink is less pronounced in the dark-state protein embedded in the cell wall due to lateral pressure. Consequently, the straightening of the helix would be less of an overall change, resulting in smaller spectral signatures. A similar effect has been observed in a soluble protein, not caused by membrane tension but by molecular crowding, which limits structural changes.^52^ On the other hand, the confined space of the protein could also affect the dynamics of the structural changes, thus leading to a different mixture of intermediates trapped at 11 ms. Consequently, it is not clear whether the differences between the spectra in the amide I region are solely due to a varying mixture of intermediates or if the spatial constraint leads to different secondary structure changes. In the future, the amide I region will be studied at a higher time resolution to answer these questions. In order to conduct these experiments, the SNR needs to be further improved.

To obtain more detailed information about the dynamics of the photocycle concerning changes in the retinal region, kinetic traces were recorded with nanosecond time resolution using a home-built QCL setup in the region from 1560 to 1516 cm^–1^. The higher intensity of the QCL light source allowed the use of thicker samples and, therefore, the detection of very small signals.

Kinetic traces in the range 1560–1516 cm^–1^ were measured for both the detergent-solubilized and the cell samples (Figure 4). Here, the negative signal, reflecting the bleach of the retinal C=C stretching vibration after photoexcitation, is shifted by 4 cm^–1^ between the samples. This is in agreement with the data from the rapid scan experiments and indicates different salt concentrations in the samples, possibly originating from slight differences in the preparation procedures. However, the overall photocycle shows a strong resemblance in both samples, judging by the similarity of the spectral features at 1 μs and 1 ms. Overall, in both data sets, the band patterns are in very good agreement despite the spectral shift. This is further supported by the kinetics of the O state monitored at 1520 cm^–1^. The kinetic trace is not strongly affected as shown by the time constants for the rise (0.4 and 0.5 ms) and decay (10 and 20 ms), respectively. Again, the differences in the decay time constants might arise from different NaCl concentrations in the samples. Considering the overall strong similarities between the spectral features of the samples, it can be concluded that the environment of the cell membrane induces only minor changes in the dynamics of the photocycle. To get a clearer picture of the influence of the cell membrane on the secondary structural changes, the sample preparation has to be further improved to allow studies in the amide I region using QCLs.

**Figure 6:**
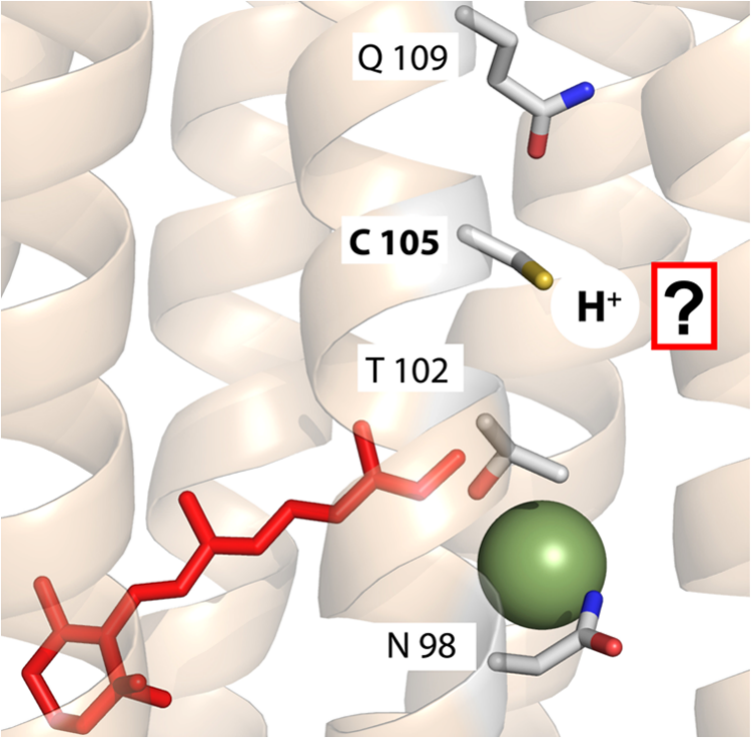
Structure of the active site of *Nm*HR (PDB: 5G28(16)). The dark-state structure is shown with the retinal (red) and the chloride ion (green). Residues N98, T102, and Q109 (NTQ motif), which were identified as essential for chloride transport, are depicted as sticks and are labeled. Before 75 ns, the cysteine residue gets deprotonated and the proton is transferred to a so far unidentified acceptor.

At 2550 and 2548 cm^–1^, a spectral region typical for the S–H stretching vibration of cysteines,^53^ a negative band at 11 ms, is detected in both samples via rapid scan FTIR spectroscopy, indicating a deprotonated cysteine residue (Figure 3, inset). The cysteine residue 105 close to the conserved NTQ motif was identified as the reactive amino acid by mutational studies. The data revealed that deprotonation occurs within 75 ns close to the limits of the time resolution of the instrument (Figure 5). Consequently, kinetic information for the rise of the signal could not be extracted. Strikingly, a recent time-resolved X-ray free electron lasers (XFEL) study of *Nm*HR did not detect any changes in the hydrogen bonding of the SH group of this residue during the photocycle.^15^ It is possible that the light-induced changes in the XFEL study were not strong enough to show up in the difference ion electron density. Interestingly, in the crystal structure of *Nm*HR, the SH group of C105 does not form hydrogen bonds with neighboring residues, leaving the question of potential hydrogen acceptors unanswered (Figure 6).^16,54^ It could be speculated that the proton is instead transferred to the backbone CO group of the cysteine itself or to a neighboring amino acid such as Ala101, which is only 3.6 Å away. It has been previously observed that the SH group of cysteines are prone to form hydrogen bonds to backbone OH-groups.^55^

The signal in both samples in our FTIR data shifts to lower frequencies at later time points, indicating inhomogeneity in the sample, possibly due to two rotamers with respect to the C–S–H torsion.^32^ The quality of the data from the cell sample allowed analysis of the band by peak-fitting. Here, two species were found, one predominantly present at 75 ns with a maximum frequency found at 2555 cm^–1^ and another species with a maximum at 2549 cm^–1^, predominantly present at 1 ms. This highlights the power of the method to determine the environment and rotamere composition of a single amino acid. These results are particularly relevant to studies of intracellular processes, such as redox homeostasis, representing a key factor in the regulation of cell signaling, development, health, and disease. Furthermore, the ability to track minute changes of single cysteine residues in living cells allows studying the response of cells to oxidative stress and changes in the overall thiol–disulfide redox balance via thiol-based regulatory switches.^20^ Here, the observation of the dynamics of the formation and breakage of disulfide bonds gives insight into in vivo redox sensing. Due to the sensitivity to its immediate environment, the frequency of the S–H vibration of the cysteines band gives insight into the hydrogen bonding scenario of the amino acid side chain. The more the band is shifted to lower frequencies, the more strongly the residue is hydrogen-bonded,^43^ providing detailed insight into the molecular environment of the residue.

The detection of the signatures of individual amino acids in a cellular environment with nanosecond time resolution also highlights the continuing relevance of time-resolved IR spectroscopy even in the light of emerging advanced techniques. Recently, time-resolved XFEL studies, time-resolved cryo-electron microscopy (cryo-EM),^56^ and several advanced super-resolution microscopy techniques^57^ have delivered groundbreaking insight into dynamic processes in biomolecules. However, label-free detection of the dynamics of single amino acids is still predominantly delivered by infrared spectroscopy due to its straightforward application and convenient sample preparation. Especially when used to study intact cells, this technique offers opportunities in the context of medical applications, ranging from optogenetics to photodynamic therapy.^58,59^ Furthermore, the extension of the method using nonphoto-induced triggers should be explored in the future, considering the ability to track small signals against strong background absorbance. We expect to record conformational changes of proteins residing in their native cellular environment at spatial resolutions that are far beyond the diffraction limit when applying the novel scanning scattering near-field optical microscopy (sSNOM).^60−62^

## Conclusions

The light response of the microbial chloride pump *Nm*HR in whole *E. coli* cells was monitored with nanosecond time resolution using transient absorption spectroscopy in the IR and UV/vis ranges. We were able to monitor the rise of the O intermediate, which is associated with chloride pumping and detected the light-induced deprotonation of a single cysteine residue taking place during chloride transport. Our methodological approach presents an excellent tool to study molecular processes on the level of single amino acid residues in the environment of whole cells with high spatiotemporal resolution. This development gives new direction to the field of in-cell studies and optogenetics.

## Supporting Information Available

Cell count assay; test of the light response in cells; UV/vis flash photolysis data of *E. coli* cells with *Nm*HR; time-resolved UV/vis flash photolysis data of *Nm*HR overexpressed in *E. coli* cells; UV/vis absorbance spectra of *Nm*HR wild-type solubilized in detergent at indicated NaCl concentrations; time constants of the rise and decay of the O_2_ state of *Nm*HR in cells and detergent-solubilized; assignment of vibrational modes of *Nm*HR detected via FTIR difference spectroscopy; light-induced light-emitting diode (LED) difference steady-state FTIR data of *Nm*HR WT, C105S, and C55S solubilized in detergent; time-resolved IR spectroscopy on *Nm*HR wild-type and C105S overexpressed in whole cells; and spectral fits of the cysteine bands of the whole-cell sample at 75 ns and 1 ms (PDF)

## Abbreviations

*Nm*HR: *Nonlabens marinus* halorhodopsin
NTQ motif: Asn-Thr-Gln
QCL: quantum cascade laser
