# Supplementary material for: Nanosecond Transient IR Spectroscopy of Halorhodopsin in Living Cells
Source: J Am Chem Soc. 2024 Jul 1;146(28):19118–27. doi: 10.1021/jacs.4c03891 (PMC11258790; doi:10.1021/jacs.4c03891)
Supplement: Supplementary file 1 — ja4c03891_si_001.pdf [file ja4c03891_si_001.pdf]

Supplementary Information

Nanosecond transient IR spectroscopy of halorhodopsin in living cells

Sabine Oldemeyer[1]*, Mariafrancesca La Greca[2], Pit Langner[1], Karoline-Luisa Lê Công[1], Ramona Schlesinger[2], Joachim Heberle[1]*

[1]Experimental Molecular Biophysics, Department of Physics, Freie Universität Berlin, Arnimallee 14, 14195 Berlin, Germany

[2]Genetic Biophysics, Department of Physics, Freie Universität Berlin, Arnimallee 14, 14195 Berlin, Germany

Corresponding Authors

* Joachim Heberle – Experimental Molecular Biophysics, Department of Physics, Freie Universität Berlin, 14195 Berlin, Germany; orcid.org/0000-0001-6321-2615;
Email: Joachim.heberle@fu-berlin.de
* Sabine Oldemeyer – Experimental Molecular Biophysics, Department of Physics, Freie Universität Berlin, 14195 Berlin, Germany; orcid.org/0000-0001-7139-7218;
Email: sabine.oldemeyer@fu-berlin.de

Experimental Section

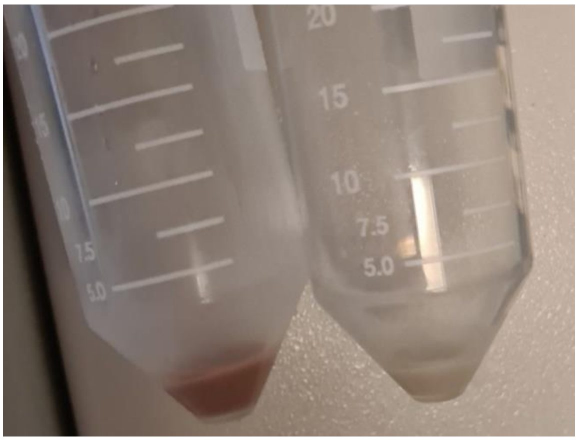

SI Fig. 1: Pelleted *E. coli* cells containing the plasmid with the *Nm*HR WT sequence with (left) or without (right) induction with 0.5 mM IPTG and the addition of 12.5 µM *all-trans* retinal. The cells overexpressing *Nm*HR WT shows an intense purple color whereas the non-induced cell pellet of protein is white.

Cell count assay

Cell viability before and after the experiment was assessed by fluorescence spectroscopy using the BacLight viability kit. This essay allows to differentiate between cells with an intact cell wall and those with a permeable membrane, based on a dual staining procedure using

SYTO 9 and propidium iodide. Cells with a permeable cell wall considered dead[1-3]. First, cells were compared before and after desiccation for 30 min and constant LED illumination at 535 nm for 10 min. The untreated cells showed an average of 10% dead cells, whereas the percentage of dead cells after the treatment was 21 (Fig. 2). This shows that the vast majority of cells are still alive after the reduction of the water content and illumination. In a second experiment, untreated cells were compared to cells with reduced water content and 1.5 hours of pulsed laser irradiation (λ = 532 nm, E_{exc}/A = 3 mJ/cm^2). In this experiment, 36% of the cells died during the experiment. It can therefore be concluded that the data were obtained from a sample with a predominance of live cells.

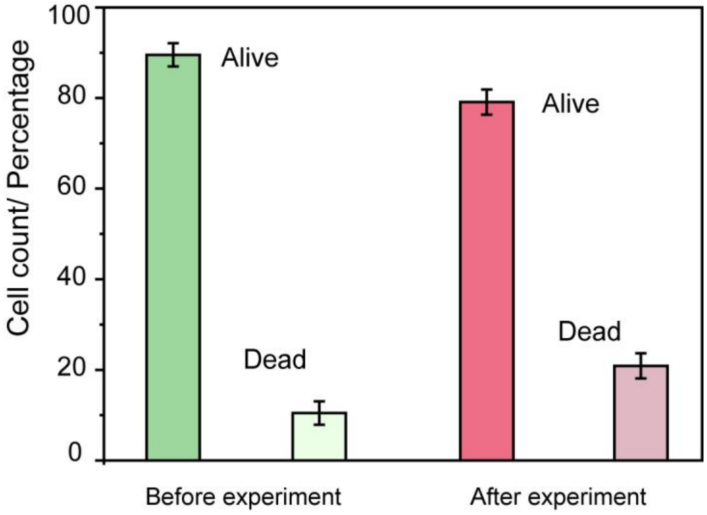

SI Fig. 2: Block diagram of the results of the BacLight cell count assay.

Test of the light-response in cells

As a control experiment, whole cell samples, prepared with a reduced extracellular water content, with and without induction via IPTG, were excited at 532 nm. Kinetic traces detected at 650 nm, indicative of the O state, were taken. The cells in which the expression of *Nm*HR was not induced did not show a signal, whereas the induced cells showed the characteristic rise and decay of the O state at 650 nm with time constants of τ_{rise}=0.9 ms and τ_{decay}=4 ms, similar to literature data[4] where time constants of 0.4 and 5 ms were derived from kinetic

analysis (SI Fig.3). This confirms that the observed spectral changes originate from the overexpressed protein and no other cellular components.

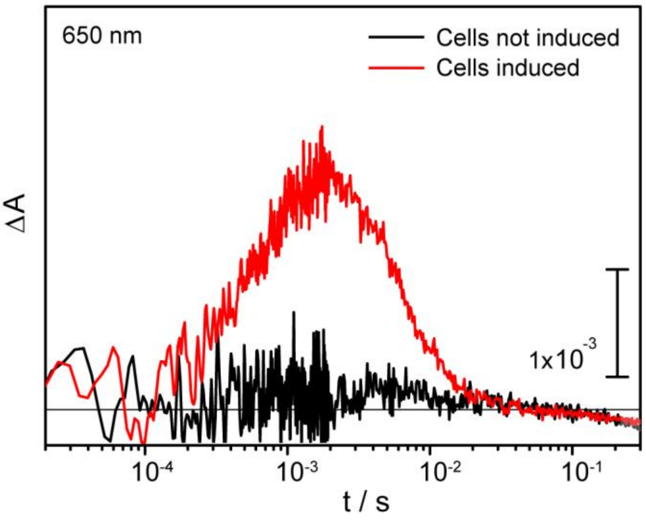

SI Fig. 3: UV-Vis flash-photolysis data of *E. coli* cells transformed with the plasmid containing the *Nm*HR wild type gene with (red) and without (black) induction. Kinetic traces were recorded at 650 nm, following the dynamics of the O state. The cells from the cultures to which 50 mM of IPTG and 12.5 µM of *all-trans* retinal were added during expression, show the characteristic rise and decay of the O state at 650 nm. The cells of the cultures which were not induced during expression do not show a signal, indicating that the observed kinetic trace originates from over-expressed *Nm*HR.

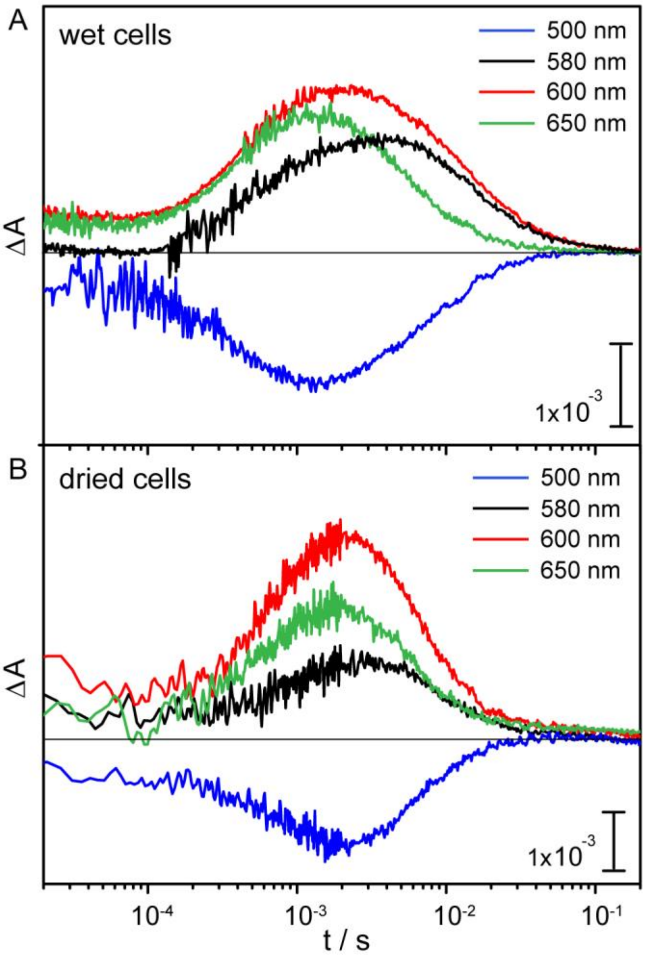

SI Fig. 4: Time-resolved UV-Vis flash-photolysis data of *Nm*HR overexpressed in *E.coli* cells detected at wavelength indicative of relevant intermediate states in different preparations. (A) Around 15 µL of the cell suspension was spread out on a BaF_2 window and immediately sealed with a second window. (B) Again, 15 µL of the cell suspension was placed on a BaF_2 window and the water content was gently reduced at atmospheric pressure by not sealing it with a second window for 15 min.

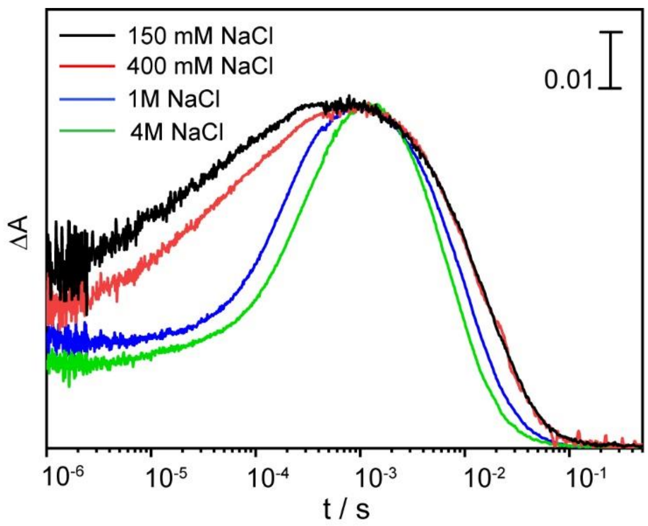

SI Fig. 5: Time-resolved UV-Vis flash photolysis data of *Nm*HR wild type solubilized in detergent with different salt concentrations (indicated) at pH 7.5 detected at 600 nm, indicative of the O state. The traces at 150 and 400 mM NaCl were scaled by factor of 1.95 and 1.55, respectively.

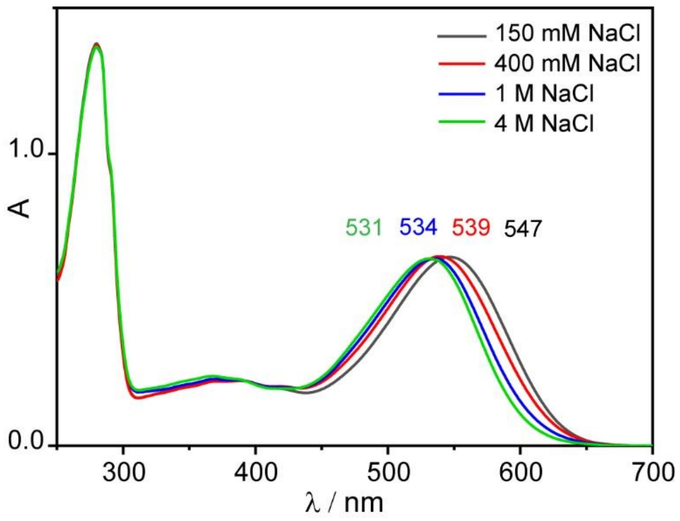

SI Fig. 6: UV-Vis absorbance spectra of *Nm*HR wild type solubilized in detergent at indicated NaCl concentrations at pH 7.5 and 20 mM HEPES.

SI Table 1: Time constants of the rise and decay of the O2 state of *Nm*HR in cells and detergent-solubilized, under different conditions, monitored at 600 nm. Literature values[4] are given in parentheses.

Condition	τ_{rise} O_1-O_2 (ms)	τ_{decay} O_2-*Nm*HR* (ms)
Cells 150 mM NaCl	0.5	16.1
Cells semi-dried	1.0	6.1
Detergent 150 mM NaCl	-	18.3
Detergent 400 mM NaCl	- (5)	17.9 (15)
Detergent 1M NaCl	0.3 (5)	11.8 (8)
Detergent 4M NaCl	0.4 (4)	8.3 (5)

Band assignment of FTIR data in the range from 1800-1100 cm^{-1}.

The band pattern at 1666 (-) and 1658 (+) cm^{-1} reflects changes in the amide I modes of the protein backbone, indicative of changes in the α-helical structure that accompanies chloride transport. At 1643 cm^{-1}, a prominent negative band with a positive counterpart at 1626 cm^{-1} is detected, possibly assignable to the C=N-H stretching mode of the Schiff base as observed via resonance Raman spectroscopy on the conversion of the ground to the O intermediate in *Nm*HR, *Hs*BR, and *Hs*HR [5-11]. The vibrational modes at 1533 (-) and 1516 cm^{-1} (+) are assigned to the change in frequency of the C=C stretching vibration of the retinal from ground state to the O state in accordance with a shift of the maximum in the electronic absorption from 535 nm to 620 nm[12]. The positive bands at 1298 and 1386 cm^{-1} can be assigned to the N-H and C_{15}-H in-plane bending vibrations of 13-*cis* retinal, respectively. The bands at 1203 (-) and 1188 cm^{-1} (+) represent the C-C stretching modes of the retinal that respond to the formation of 13-cis retinal in the presence of a protonated Schiff base.

SI Table 2: Assignment of vibrational modes of *Nm*HR detected via FTIR difference spectroscopy

***Nm*HR cells**		**References**			**Assignment**
**negative**	**positive**	***Nm*HR detergent**	**BR[13]**	***Np*HR[14]**	
1690		1697[15]		1694	Asn ν_{as} $CN_3H_5^+$
1664		1666[16]	1671	1674	α-helix
	1657	1658[16]	1663	1657	α-helix
1639		1643[16]	1639		ν C=N-H Schiff base
	1623	1626	1628	1630	ν C=N-H
1537		1533	1526	1525	ν C=C retinal
	1520	1516	1506	1510	ν C=C retinal
	1394	1388			δ C_{15}-H in-plane retinal
	1327	1331			
	1300	1298			δ N-H
1203				1209	ν C-C retinal
	1188	1887	1185	1195	ν C-C retinal

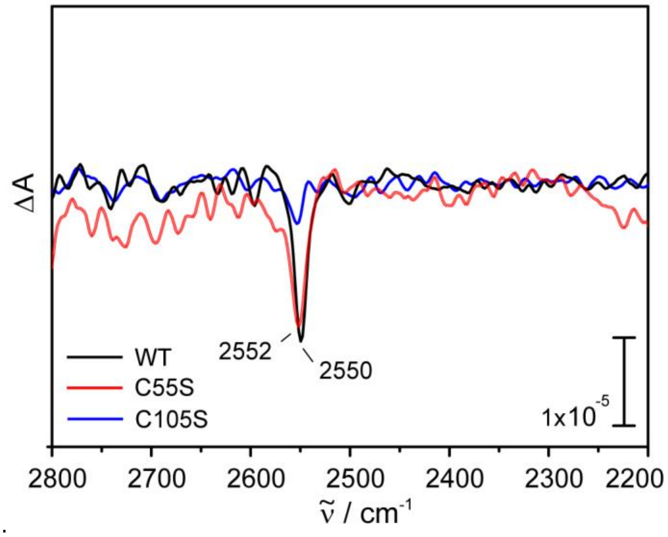

SI Fig. 7: Light-induced LED difference steady state FTIR data of *Nm*HR wild type, C105S and C55S solubilized in detergent covering the spectral range from 2800 to 2200 cm^{-1}. Samples were illuminated for 1 second at 525 nm with an LED. 4 µl of the concentrated protein solution (~ 30-40 mg/ml) in 150 mM NaCl, 20 mM HEPES at pH 7.5 with 0.03% DDM were dried on a BaF_2 window under dried air and subsequently rehydrated with 5 µl of a mixture of H_2O and glycerol (50/50 v/v) for 2 hours. A spacer and a second BaF_2 window were placed on top to prevent the samples to dry during the experiment. The measurements were accomplished using a Vertex 80V FTIR spectrometer in transmission mode with a spectral resolution of 4 cm^{-1}. The light-induced LED difference spectra were carried out subtracting the spectra in the dark and under green light (LED: 525 nm). For each protein 30000 scans were averaged to achieve a good signal to noise ratio.

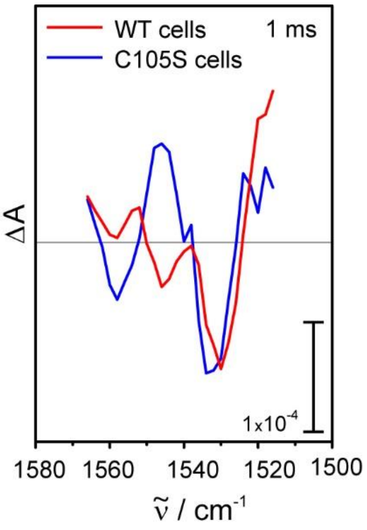

SI Fig. 8: Time-resolved IR spectroscopy on *Nm*HR wild type (red) and C105S (blue) overexpressed in whole cells. Spectra extracted at 1 ms upon the maximum accumulation of the O-state show for both samples a light-response as indicated by the negative band at 1538 cm^{-1}. The C105S sample was averaged 300 times for each wavenumber with a spacing of 2 cm^{-1}.

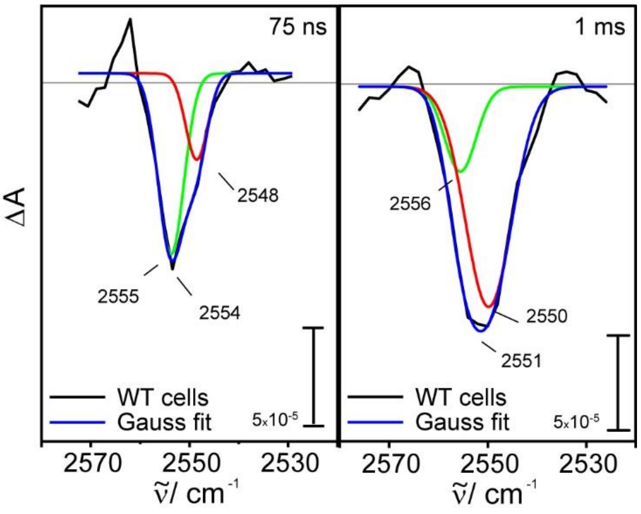

SI Fig. 9: Gaussian fit of the cysteine bands of the whole cell sample at 75 ns and 1 ms. At both time points, the band can be fitted with two Gaussians (green and red) with maxima at 2555/2556 cm^{-1} and 2548/2550 cm^{-1}. The significant differences in proportion between the two components at 75 ns and 1 ms indicate the presences of two rotamers with different hydrogen bonding scenarios resulting in differing deprotonation dynamics.

REFERENCES

1. Robertson, J.; McGoverin, C.; Vanholsbeeck, F.; Swift, S., Optimisation of the Protocol for the LIVE/DEAD(®) BacLight(TM) Bacterial Viability Kit for Rapid Determination of Bacterial Load. *Frontiers in microbiology* **2019,** *10*, 801.
2. Berney, M.; Hammes, F.; Bosshard, F.; Weilenmann, H. U.; Egli, T., Assessment and interpretation of bacterial viability by using the LIVE/DEAD BacLight Kit in combination with flow cytometry. *Applied and environmental microbiology* **2007,** *73* (10), 3283-90.
3. Stiefel, P.; Schmidt-Emrich, S.; Maniura-Weber, K.; Ren, Q., Critical aspects of using bacterial cell viability assays with the fluorophores SYTO9 and propidium iodide. *BMC microbiology* **2015,** *15*, 36.
4. Tsukamoto, T.; Yoshizawa, S.; Kikukawa, T.; Demura, M.; Sudo, Y., Implications for the Light-Driven Chloride Ion Transport Mechanism of Nonlabens marinus Rhodopsin 3 by Its Photochemical Characteristics. *The journal of physical chemistry. B* **2017,** *121* (9), 2027-2038.
5. Smith, S. O.; Myers, A. B.; Pardoen, J. A.; Winkel, C.; Mulder, P. P.; Lugtenburg, J.; Mathies, R., Determination of retinal Schiff base configuration in bacteriorhodopsin. *Proc Natl Acad Sci U S A* **1984,** *81* (7), 2055-9.
6. Alshuth, T.; Stockburger, M.; Hegemann, P.; Oesterhelt, D., Structure of the retinal chromophore in halorhodopsin: A resonance Raman study. *FEBS Letters* **1985,** *179* (1), 55-59.
7. Aton, B.; Doukas, A. G.; Narva, D.; Callender, R. H.; Dinur, U.; Honig, B., Resonance Raman studies of the primary photochemical event in visual pigments. *Biophysical journal* **1980,** *29* (1), 79-94.
8. Baasov, T.; Friedman, N.; Sheves, M., Factors affecting the C = N stretching in protonated retinal Schiff base: a model study for bacteriorhodopsin and visual pigments. *Biochemistry* **1987,** *26* (11), 3210-7.
9. Rothschild, K. J., FTIR difference spectroscopy of bacteriorhodopsin: Toward a molecular model. *Journal of bioenergetics and biomembranes* **1992,** *24* (2), 147-167.
10. Rothschild, K. J.; Bousché, O.; Braiman, M. S.; Hasselbacher, C. A.; Spudich, J. L., Fourier transform infrared study of the halorhodopsin chloride pump. *Biochemistry* **1988,** *27* (7), 2420-4.
11. Ohya, M.; Kikukawa, T.; Matsuo, J.; Tsukamoto, T.; Nagaura, R.; Fujisawa, T.; Unno, M., Structure and Heterogeneity of Retinal Chromophore in Chloride Pump Rhodopsins Revealed by Raman Optical Activity. *The Journal of Physical Chemistry B* **2023,** *127* (21), 4775-4782.
12. Aton, B.; Doukas, A. G.; Callender, R. H.; Becher, B.; Ebrey, T. G., Resonance Raman studies of the purple membrane. *Biochemistry* **1977,** *16* (13), 2995-9.
13. Zscherp, C.; Heberle, J., Infrared difference spectra of the intermediates L, M, N, and O of the bacteriorhodopsin photoreaction obtained by time-resolved attenuated total reflection spectroscopy. *J. Phys. Chem. B* **1997,** *101*, 10542-10547.
14. Hackmann, C.; Guijarro, J.; Chizhov, I.; Engelhard, M.; Rödig, C.; Siebert, F., Static and time-resolved step-scan Fourier transform infrared investigations of the photoreaction of halorhodopsin from *Natronobacterium pharaonis*: consequences for models of the anion translocation mechanism. *Biophys. J.* **2001,** *81* (1), 394-406.
15. Iwata, T.; Zhang, Y.; Hitomi, K.; Getzoff, E. D.; Kandori, H., Key Dynamics of Conserved Asparagine in a Cryptochrome/Photolyase Family Protein by Fourier Transform Infrared Spectroscopy. *Biochemistry* **2010,** *49* (41), 8882-8891.
16. Mous, S.; Gotthard, G.; Ehrenberg, D.; Sen, S.; Weinert, T.; Johnson, P. J. M.; James, D.; Nass, K.; Furrer, A.; Kekilli, D.; Ma, P.; Brünle, S.; Casadei, C. M.; Martiel, I.; Dworkowski, F.; Gashi, D.; Skopintsev, P.; Wranik, M.; Knopp, G.; Panepucci, E.; Panneels, V.; Cirelli, C.; Ozerov, D.; Schertler, G. F. X.; Wang, M.; Milne, C.; Standfuss, J.; Schapiro, I.; Heberle, J.; Nogly, P., Dynamics and mechanism of a light-driven chloride pump. *Science* **2022,** *375* (6583), 845-851.
